# Current evidence on patient precautions for reducing breast cancer-related lymphedema manifestation and progression risks

**DOI:** 10.1007/s12032-024-02408-3

**Published:** 2024-10-17

**Authors:** Julie Hunley, David Doubblestein, Elizabeth Campione

**Affiliations:** 1 Department of Occupational Therapy, Mount Mary University, Milwaukee, WI USA; 2 Department of Physical Therapy, A.T. Still University, Mesa, AZ USA; 3 Physical Therapy Program, Midwestern University, Downers Grove, IL USA

**Keywords:** Lymphedema, Air travel, Blood pressure, Surgery, Temperature, Blood draws, Trauma, Behavior

## Abstract

Risk management and self-management strategies for breast cancer-related lymphedema (BCRL) must balance best-evidence guidelines and associated risk factor knowledge. There is an evidence-based practice gap in the understanding of whether a change in education about risk factors and whether behavioral changes actually influence BCRL manifestation or progression. The purpose of this study was to (1) review if current evidence supports or refutes patient precautions to prevent the manifestation and/or progression of BCRL, (2) review if behavioral changes result in the prevention or reduction of BCRL, and (3) identify related gaps of knowledge for future research. Evidence map methodology was used to systematically review literature related to reducing the risk of BCRL. Literature searches were conducted in Medline, CINAHL, and Cochrane for the categories of trauma, blood pressure, temperature, air travel, and behavior change. One hundred and forty-eight articles were included for full-text review, of which 37 articles were included in this study. Within the confines of limb and trunk circumferential and/or volume enlargement, a ‘just in case’ approach to patient education on risk factors may not be appropriate for breast cancer survivors at risk of manifesting lymphedema. Patient education about precautionary risks for the onset of BCRL needs to align with research evidence. There is scant evidence about the risks of BCRL progression suggesting a need for future research.

## Background

Approximately, 4 million breast cancer survivors are living in the United States [1]. Advances in prevention, diagnosis, and treatment have contributed to this sizable survivorship. This long-term survival may effectuate the treatment side-effect of breast cancer-related lymphedema (BCRL) manifested in the ipsilateral upper quadrant. While breast cancer survivors incur a lifetime risk of developing this incurable condition, with a pooled incidence of 21.9% (95% CI 19.8–24.0%) [2], it is purported to be preventable and certainly manageable [3, 4]. A goal for both practitioner and patient is the prevention of the manifestation or progression of BCRL, which involves the surveillance of risk factors across the continuum of care. The recent inception of post-operative prospective surveillance procedures along with self-management behaviors such as skin care, compression, elevation, therapeutic exercises, and self-administered lymphatic drainage is deemed a continuum of care that may reduce the risk of BCRL manifestation [5–7]. Those who have BCRL may continue these measures to avoid the progression of the condition.

Risk factors can be branched into treatment-related and patient-specific risk exposures. Treatment-related risk factors are only adjusted or avoidable prior to the intervention and include lymphadenectomy, chemotherapy, mastectomy, irradiation of lymph nodes, and post-operative complications [2]. While current guidelines for these procedures have decreased the associated incidence of BCRL, these risk factors continue to be significant contributors to its manifestation [2, 8–11]. Relative volume change is a significant risk factor [2, 3] with a > 5% increase in relative volume change 1 month post-operatively having an odds ratio of 5.54 (95% CI 0.72–7.9) [2].

Patient-specific risk exposures are modifiable by risk management behaviors. Historically, moderate and high levels of exercise were considered a risk for BCRL, but research over the last decade has proven otherwise and the promotion of physical activity in breast cancer survivors is rightly promoted [12, 13]. Elevated body mass index (BMI) is a significant risk factor for the manifestation and progression of BCRL. Both pre-operative BMI and post-operative weight increase have been accounted as significant risk factors for the manifestation and exacerbation of BCRL [2, 3, 14]. A baseline BMI of ≥ 30 kg/m^2^ prior to breast cancer treatment is a risk factor for BCRL [15], as is a weight gain of 5 kg post-operatively can increase the risk of BCRL by 8.8% [2]. Cellulitis is an established significant risk factor [3, 14] that can both manifest and exacerbate BCRL and to some extent is modifiable via prophylactic skin care. Other patient-specific risk exposures include air travel, ipsilateral blood pressure screening, skin punctures (e.g., blood draws, injections), surgeries, and traumas [2, 8, 11, 16, 17]. For several decades, these risk factors have been a part of education provided to breast cancer survivors to make behavioral changes to minimize the risk of lymphedema. Patient-specific risk management behaviors have also been propagated through various societies such as the National Lymphedema Network [18], American Cancer Society [19], National Cancer Institute [20], and the Susan G. Komen Organization [21]. Despite their acclaim, many of these risk factors have been unsubstantiated [22], based on anecdotal case observations and associated pathophysiology [23], and have rightly been scrutinized in recent literature [2, 8, 11, 17].

Authors have claimed that there is fear with regard to lymphedema [3, 24, 25] and concern that the promulgation of BCRL risk factors may induce avoidance behaviors, fear, anxiety, or frustration among breast cancer survivors [2, 14, 22, 23]. This fear of BCRL may actually be rooted pre-operatively and extend beyond 2 years post-operatively [25]. Jammallo et al. [25] claimed that the fear of lymphedema manifestation is actually greater than the fear of its progression. In contrast, Uhlmann et al. [26] claimed that despite the limited knowledge of BCRL risk factors, most breast cancer survivors were not worried about BCRL manifestation. Higher worry was associated with a higher BCRL stage, axillary lymphadenectomy, and employment [26]. There is evidence that greater adherence to risk management strategies is associated with greater knowledge, perceived self-regulatory ability, and controllability of lymphedema, rather than the perceived consequences [16]. Yet, apart from skincare and actively avoiding injury and infection, adherence to self-management behaviors ranges from 65.0 to 71.1% [27]. Adherence was also negatively associated with levels of lymphedema distress (*r* = − 0.15, *p* = 0.052) [27].

Risk management and self-management strategies must balance best-evidence BCRL guidelines and associated risk factor knowledge with catalysts that foster adherence to surveillance and preventative behavioral strategies. Unfortunately, an evidence-based practice gap remains in what is best available evidence, clinical expertise in the delivery of the evidence, patient psychosocial perspectives, and the social context that envelops this patient-centered care. Best available evidence on patient-specific risk exposures and precautions related to BCRL is scarce and is likely due to the paucity of funding for robust high-level research designs. There is a gap of understanding about whether a change in dialog about risk factors and whether behavioral changes actually influence BCRL manifestation or progression. To that end, the purpose of this study was to (1) review if current evidence supports or refutes patient precautions for the purpose of preventing the manifestation and/or progression of BCRL, (2) review if behavioral changes result in the prevention or reduction of BCRL, and (3) identify related gaps of knowledge for future research.

## Methods

### Study design

Evidence map methodology was used to systematically review literature related to reducing the risk of BCRL. Evidence maps are highly accessible descriptive representations of the gaps in evidence in a broad field [28].

### Eligibility criteria

Studies were eligible for inclusion if (1) they were intervention, prospective, retrospective, or case study designs; (2) included adult breast cancer survivors with or without BCRL; (3) studied patient-specific risk exposures associated with BCRL in the upper quadrant ipsilateral to breast cancer or interventions focused on risk reduction self-care management strategies; and (4) included objective measurements of lymphedema. Eligible research focused on the relationship of BCRL and treatment-related factors like lymphadenectomy, chemotherapy, mastectomy, irradiation of lymph nodes, and post-operative complications. Studies were excluded if they included primary lymphedema or secondary lymphedema unrelated to breast cancer populations. Secondary analyses (e.g., meta-analyses, systematic, and other reviews) and expert opinion designs were excluded from this review.

### Search strategy

A search strategy was developed using keywords and Medical Subject Headings (MeSH) that captured the eligibility criteria. Anatomical keywords included arm, hand, breast, upper extremity, and trunk. Patient-specific risk factor keywords included trauma (e.g., fracture, wound, surgery), air travel, medical punctures (e.g., injection, blood draw, infusion), blood pressure measurement, and risk reduction self-care management. Objective lymphedema measurement was the primary outcome of interest. Medline, CINAHL, and Cochrane databases were searched between January 2008 and March 2023. Hand searches of systematic review reference lists were completed to ensure saturation. No gray literature search was conducted.

### Study selection and extraction

**Fig. 1:**
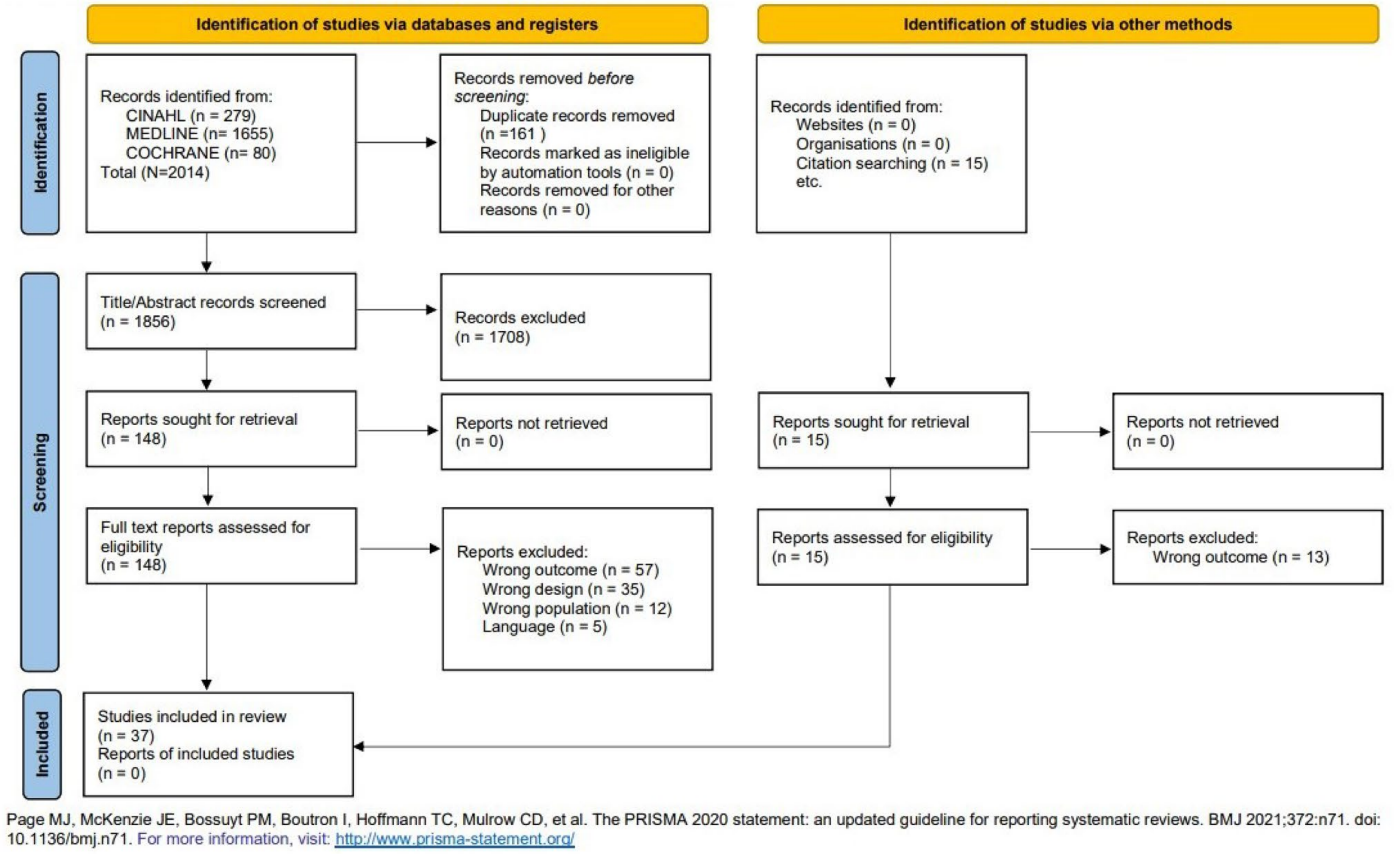
PRISMA flow diagram

Studies returned by the database searches were retrieved by title and abstract and uploaded into Rayyan [29]. Duplicates were removed, and each title and abstract were reviewed by two independent reviewers. Conflicts between reviewers were discussed and resolved or a third reviewer resolved the disagreement. Full-text screening was completed using the same process of two independent reviewers with a third reviewer available to resolve conflicts. A PRISMA flowchart in Fig. 1 illustrates the selection process.

Final full-text databases were created, and data extraction ensued. Data were extracted for each study by two independent reviewers. Extracted data included author, year, study type, level of evidence, confidence level, sample size, diagnosis, independent variable, and outcomes. In studies on patient-specific risk exposures, the sample size represented the portion of the sample with the risk exposure rather than the entire sample. The level of evidence was assigned to each study [30]. After comparing data extraction results, conflicts were resolved by the original two reviewers or with the assistance of the third reviewer.

### Data synthesis and analysis: evidence maps

Studies were synthesized according to risk reduction intervention or the patient-specific risk exposure categories of trauma, blood pressure, temperature, and air travel. An estimate of confidence was determined using methods referenced by Kondo et al. [31] and described by Miake-Lye, et al. [28] and Hempel, et al. [32] Confidence calculations started with the level of evidence and added points as follows; the included study design (prospective +1), statistical adjustment for confounding variables (+1), multisite (+1), and large sample (+1) [31]. Confidence levels ranged from 1 (low) to 6 (high). Studies were grouped by diagnostic category (at-risk for or diagnosed with BCRL) and BCRL outcome (manifested, exacerbated, not manifested, not exacerbated, or unclear). Unclear outcome representation occurred when study samples included both populations (those at risk for manifestation of BCRL and those already diagnosed with BCRL) without separate statistical analysis of the outcomes by diagnosis. Evidence bubble maps were created for each risk category and intervention by plotting studies on a graph with the *x*-axis representing BCRL outcomes as not manifested, unclear, not progressed, or manifested, unclear, progressed. Patient-specific risk events within a risk category were plotted on the *y*-axis. The estimate of confidence is illustrated by the size and color of the bubble.

## Results

### Study selection

The initial search in Medline, CINAHL, and Cochrane yielded 2014 total articles for the categories of trauma, blood pressure, temperature, air travel, and behavior change. After removing duplicates, 1856 titles and abstracts were screened by two independent reviewers. One hundred and forty-eight articles were included for full-text review by two independent reviewers. Disagreements between the two reviewers, whether during abstract or full-text reviews, were addressed through discussion focused on the inclusion and exclusion criteria. When consensus could not be met, a resolution was sought from a third reviewer. Thirty-seven articles were included in this study (PRISMA Fig. 1).

### Evidence map of trauma as a risk factor

**Fig. 2:**
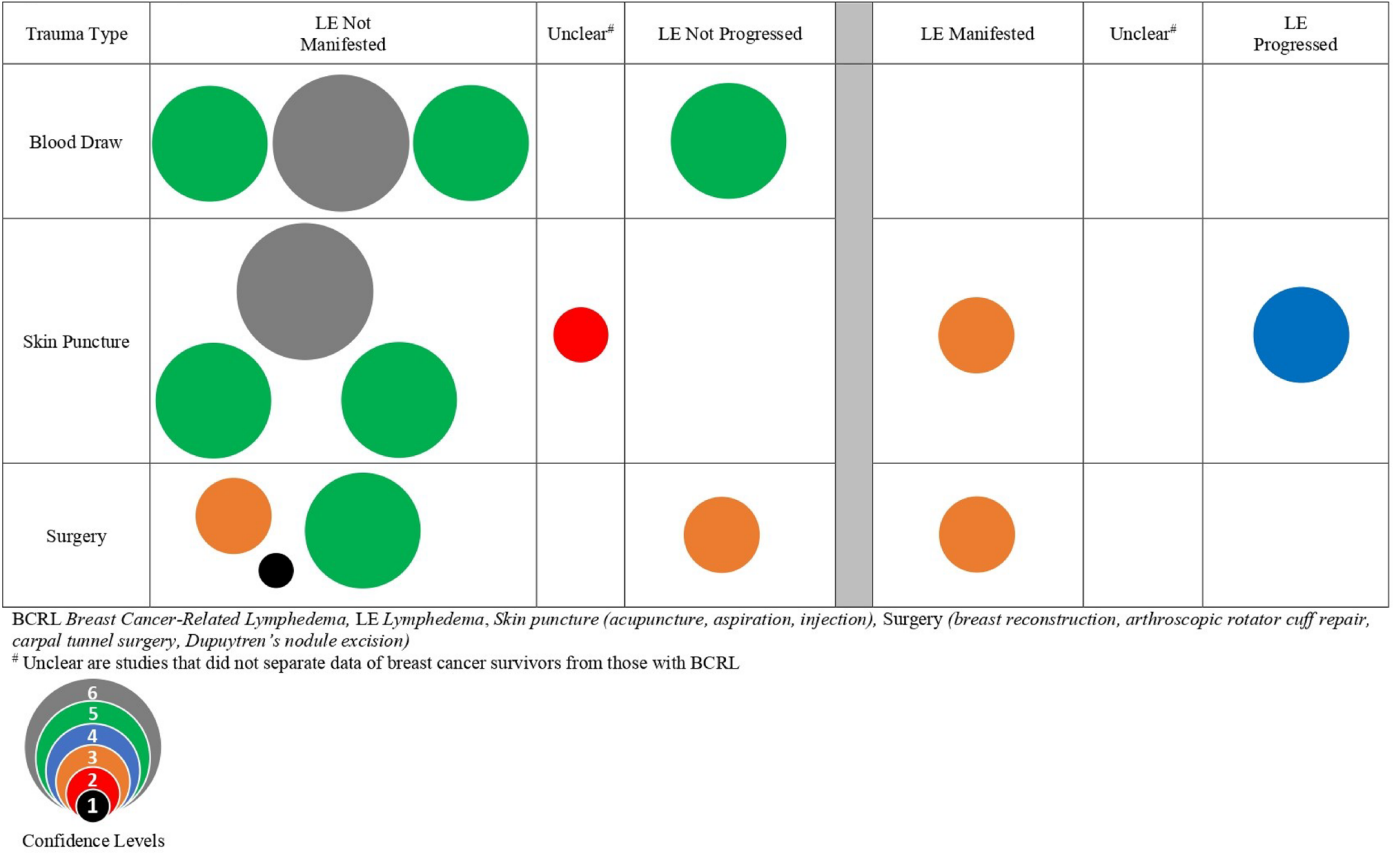
Precautionary risk of trauma on breast cancer survivors and BCRL

The results for trauma to the ipsilateral quadrant as a risk factor for the manifestation or progression of BCRL are presented in the bubble map (Fig. 2) and Table 1. The bubble map synthesizes 10 studies relevant to traumas including blood draws, skin puncture, and surgery. Table 1 summarizes 17 studies relevant to traumas including blood draws, skin punctures, surgery, seromas, other trauma events, heavy exercise, and wound infection.

**Table 1:** Trauma study characteristics and outcomes

Author (year)	Study type; level of evidence; confidence level	Sample size, diagnosis	Independent variable	Outcomes
Asdourian et al. (2016) [11]	Prospective cohort; Level 4; Level 5	*N* = 209 at risk for BCRL	Blood draw	Univariate analysis: no significant association between increased volume change and ≥ 1 blood draw versus no blood draws (*p* = 0.4906)
Ferguson et al. (2016) [17]	Prospective cohort; Level 4; Level 5	*N* = 251 at risk for BCRL	Blood draw	Univariate analysis: no significant association between arm volume change and undergoing ≥ 1 blood draw (*p* = 0.62) – When analyzed as a continuous variable, number of ipsilateral blood draws were not associated with an increased arm volume (*p* = 0.92) – In patients who underwent ALND, blood draws (*p* = 0.26) were not associated with increased arm swelling Multivariate analysis: blood draws were not significantly associated with arm volume change (HR 0.977, *p* = 0.91)
Kilbreath et al. (2016) [8]	Prospective cohort; Level 4; Level 6	*N* = 112 at risk for BCRL	Blood draw	Univariate analysis/ROC: blood drawn from the at-risk limb was retained as a risk factor for lymphedema (OR 2.0, 95% CI 0.8–5.2, *p* = 0.17)
Alem et al. (2008) [33]	Case series; Level 1; Level 2	*N* = 6 at risk for BCRL *N* = 23 with BCRL	Skin puncture (acupuncture)	Multivariate analysis: no change or improvement in limb volume measurements
Asdourian et al. (2016) [11]	Prospective cohort; Level 4; Level 5	*N* = 120 at risk for BCRL	Skin puncture (injections)	Univariate analysis: no significant association was found between an increased volume change and having ≥ 1 injections versus no injections (*p* = 0.0928)
Clark et al. (2004) [34]	Prospective cohort; Level 3; Level 3	*N* = 18 at risk for BCRL	Skin puncture (hospital skin puncture)	Relative risk: 44.4% (*n* = 8) of those who had any type of hospital skin puncture developed lymphedema (OR 2.44, 95% CI 1.33–4.47)
Ferguson et al. (2016) [17]	Prospective cohort; Level 4; Level 5	*N* = 63 at risk for BCRL	Skin puncture (injections)	Univariate analysis: no significant association between an increased arm volume change and injections in the at-risk limb (*p* = 0.77) – When analyzed as a continuous variable, injections were not associated with an increase in limb volume (*p* = 0.85) – In patients who underwent ALND, injections (*p* = 0.35) were not associated with increased arm swelling Multivariate analysis: injections were not significantly associated with arm volume change (HR 1.101, *p* = 0.5)
Kilbreath et al. (2016) [8]	Prospective cohort; Level 4; Level 6	*N* = 112 at risk for BCRL	Skin puncture (injections)	Univariate analysis/ROC: injections to the at-risk limb were not retained as a risk factor for lymphedema (OR 1.0, 95% CI 0.3–2.7, *p* = 0.92)
Swenson et al. (2009) [35]	Case control; Level 2; Level 4	*N* = 13 at risk for BCRL *N* = 28 with BCRL	Skin puncture (fluid aspiration)	Univariate analysis: patients with BCRL were more likely than those at risk to have multiple fluid aspirations after surgery (OR 1.88, *p* = 0.005) Multivariate analysis: the odds ratio decreased to 1.49 (95% CI 0.73–3.02, *p* = 0.273)
Baltzer et al. (2017) [36]	Retrospective cohort; Level 3; Level 3	*N* = 103 at risk for BCRL	Surgery (Hand surgery: CTR, trigger finger release, ganglion cyst excision, tenosynovectomy)	Descriptive statistics: 3.8% (*N* = 4) had documented lymphedema after hand surgery
Basta et al. (2015) [37]	Retrospective cohort; Level 2; Level 3	*N* = 239 at risk for BCRL or with BCRL	Surgery (breast reconstruction)	Descriptive statistics/*t* test or Wilcoxon rank sum: 19.2% of patients post-breast reconstruction developed lymphedema compared to 20.1% who did not undergo reconstruction (*p* = 0.82) – Implant-based reconstruction was associated with a 21.9% incidence of lymphedema overall, whereas autologous reconstruction demonstrated an incidence of 18.7% (*p* = 0.69) – The incidence of lymphedema was 19.7% overall, with no appreciable difference in patients who did or did not undergo breast reconstruction
Crosby et al. (2012) [38]	Retrospective cohort; Level 3; Level 5	*N* = 1013 at risk for BCRL	Surgery (breast reconstruction)	Univariate analysis: the incidence rate of BCRL was not significantly associated with reconstruction type – Patients with autologous tissue alone and latissimus dorsi myocutaneous flaps and implants had a slightly higher lymphedema incidence than did patients with expander and implant (4.31%; 4.71% versus 3.66%) Multivariate analysis: reconstruction type had no significant effect on the incidence of or time to lymphedema, and no interaction was found between axillary intervention and reconstruction type (*p* = 0.799)
Gunnoo et al. (2015) [39]	Prospective cohort; Level 3; Level 3	*N* = 32 with BCRL	Surgery (CTR)	Descriptive statistics/student’s *t* test: median lymphedema volume was 497 mL before and 582 mL after carpal tunnel surgery (*p* = 0.004)*.* At the last follow-up post-carpal tunnel surgery (median 33 months), lymphedema volume was 447 mL, a non-significant difference compared to pre-carpal tunnel surgery volume
Schwartz et al. (2020) [40]	Case study; Level 1; Level 1	*N* = 1 with BCRL	Surgery (arthroscopic rotator cuff repair)	Patient experienced post-operative edema in the arm, forearm, and wrist that resolved at the 2-week post-operative visit
Swenson et al. (2009) [35]	Case control; Level 2; Level 4	*N* = 25 at risk for BCRL *N* = 24 with BCRL	Surgery (medical procedure on arm or hand of breast cancer surgery)	Univariate analysis: surgery to the limb was not a significant variable (OR 0.94, *p* = 0.862)
Riberio-Pereira et al. (2017) [41]	Prospective cohort; Level 3; Level 5	*N* = 575 at risk for BCRL	Seroma	Multivariate analysis: patients with seroma demonstrated a higher lymphedema incidence during the follow-up period (HR 1.46, 95% CI 1.14–1.87, *p* = 0.003)
Shahpar et al. (2013) [42]	Case control; Level 3; Level 5	*N* = 27 at risk for BCRL *N* = 10 with BCRL	Seroma	Univariate analysis: seroma did not demonstrate a significant correlation with the development of lymphedema (OR 0.85, 95% CI 0.4–1.81, *p* = 0.679)
Asdourian et al. (2016) [11]	Prospective cohort; Level 4; Level 5	*N* = 52 at risk for BCRL	Trauma event(s) (bruising, fractures)	Univariate analysis: no significant association between an increased volume change and having ≥ 1 incident of trauma to the at-risk arms versus no trauma (*p* = 0.5705)
Bloomquist et al. (2021) [44]	Randomized controlled trial; Level 3; Level 3	*N* = 2 at risk for BCRL	Trauma event(s) (blunt trauma from soccer)	Descriptive statistics: no exacerbation of lymphedema or lymphedema symptoms
Ferguson et al. (2016) [17]	Prospective cohort; Level 4; Level 5	*N* = 37 at risk for BCRL	Trauma event(s) (ranged from bruising to fractures)	Univariate analysis: no significant association between increased volume change and trauma to the at-risk limb (*p* = 0.08) – In patients who underwent ALND, trauma (*p* = 0.23) was not associated with increased arm swelling Multivariate analysis: trauma was not significantly associated with arm volume change
Kilbreath et al. (2016) [8]	Prospective cohort; Level 4; Level 6	*N* = 112 at risk for BCRL	Trauma event(s) (cuts, abrasions, bites, stings, burns, sunburn, bruising, falling on at-risk limb)	Univariate analysis/ROC: trauma to the at-risk limb was not retained as a risk factor for lymphedema (OR 0.6, 95% CI 0.2–1.7, *p* = 0.33)
Li et al. (2022) [43]	Case study; Level 1; Level 1	*N* = 1 at risk for BCRL	Trauma event(s) (prolonged sun exposure)	Sunburn resulted in BCRL and cellulitis of the chest wall
Shahpar et al. (2013) [42]	Case control; Level 2; Level 3	*N* = 11 at risk for BCRL *N* = 12 with BCRL	Trauma event(s)	Univariate analysis: trauma showed a significant correlation with the development of lymphedema (OR 2.71, 95% CI 1.16–6.33, *p* = 0.02)
Showalter et al. (2013) [45]	Prospective; Level 3; Level 4	Sunburn: *N* = 56 Pet scratch: *N* = 107 Bug bite: *N* = 280 Cut: *N* = 313 Bruise: *N* = 90 Sports injury: *N* = 13 Skin burn: *N* = 26 All at risk of or with BCRL	Trauma event(s) (sunburn, pet scratch, bug bite, cut, bruise, sports injury, skin burn)	Univariate analysis: over a 12-month period, sunburns (OR 1.76, 95% CI 0.49–6.26, *p* = 0.38), pet scratches (OR 1.49, 95% CI 0.54–4.11, *p* = 0.44), bug bites (OR 1.09, 95% CI 0.49–2.45, *p* = 0.81), cuts (OR 1.99, 95% CI 0.91–4.35, *p* = 0.08), bruises (OR 1.98, 95% CI 0.69–5.67, *p* = 0.20), sports injuries (OR 1.82, 95% CI 0.35–15.12, *p* = 0.56), and skin burns (OR 2.52, 95% CI 0.53–11.93, *p* = 0.24) were not significantly associated with incident arm swelling
Swenson et al. (2009) [35]	Case control; Level 2; Level 4	*N* = 27 at risk for BCRL *N* = 10 with BCRL	Trauma event(s) (injury on arm or hand on side of surgery)	Univariate analysis: trauma was not a significant variable in the development of or exacerbation of lymphedema (OR 0.61, *p* = 0.28)
Bloomquist et al. (2014) [46]	Retrospective cohort; Level 2; Level 3	*N* = 149 at risk for BCRL	Heavy exercise with > 80% 1 repetition maximum	Descriptive statistics: no associations were found between performing heavy resistance training and the development of BCRL
Ugur et al. (2013) [47]	Prospective cohort; Level 3; Level 3	*N* = 455 with BCRL	Wound infection and lymphangitis	Descriptive statistics/Pearson chi-square: more than half (52% and 57%) of patients with wound infection (OR 3.11, 95% CI 1.41–6.82, *p* = 0.003) and lymphangitis (OR 3.83, 95% CI 1.57–9.34, *p* = 0.002) in the ipsilateral arm had lymphedema

*ALND* axillary lymph node dissection, *BCRL* breast cancer-related lymphedema, *CI* confidence interval, *CTR* carpal tunnel release, *HR* hazard ratio, *OR* odds ratio, *ROC* receiver operating characteristics

#### Blood draws

The three included studies that focused on blood draws as a risk factor for the manifestation of BCRL were prospective cohort studies [8, 11, 17] and included 572 participants at risk for BCRL. The studies did not find a significant association between arm volume change and experiencing one or more blood draws [11, 17]. However, Kilbreath et al. [8] highlighted in their study the insignificant increased odds of manifesting BCRL from a univariate analysis (OR 2.0, 95% CI 0.8–5.2, *p* = 0.17).

#### Skin puncture

Six studies were included that looked at skin puncture including injections, fluid aspirations, and acupuncture as risk factors [8, 11, 17, 33–35]. Four of the included studies were prospective cohort studies [8, 11, 17, 34], one case–control [35], and one case series [33]. Three hundred and thirty-two of the participants were at risk for BCRL and 51 had BCRL. Three [8, 11, 17] of the four studies that looked at injections did not find a significant association between limb volume and injections in the at-risk limb. Conversely, Clark et al. [34] found 44.4% (*N* = 8) of those who had a skin puncture developed BCRL (OR 2.44, 95% CI 1.33–4.47). Swenson et al. [35] found that patients with BCRL were more likely to have experienced multiple fluid aspirations after their breast cancer surgery compared to those who did not develop BCRL (OR 1.88, *p* = 0.005); however, the odds ratio decreased to 1.49 (95% CI 0.73–3.02, *p* = 0.273) when this risk factor was entered into a multivariate analysis. Acupuncture in the ipsilateral limb did not change or improve limb volume measurements in those at risk for or with BCRL; however, patients reported a subjective improvement in their degree of lymphedema after acupuncture treatment [33].

#### Surgery

Six studies looked at surgery as a risk factor for the manifestation or progression of lymphedema [35–40]. Four studies included surgery to the ipsilateral shoulder, arm, and/or hand [35, 36, 39, 40] and two studies included breast reconstruction [37, 38]. Three of the included studies were retrospective cohort studies [36–38], one prospective cohort study [39], one case-controlled, [35], and one case study [40]. Fifty-seven participants had BCRL, 1141 were at risk for BCRL, and 239 were either at risk for or had BCRL. Conflicting evidence exists as to whether surgery to the ipsilateral limb manifests or progresses lymphedema. One study found that 3.8% (4/103) of participants at risk for BCRL developed BCRL after hand surgery [36]. Two studies reported patients with BCRL had a temporary progression of their BCRL but it resolved 2 weeks post-op arthroscopic shoulder repair [40] and a median of 33 months after carpal tunnel release [39]. Conversely, Swenson et al. did not find surgery to the ipsilateral limb to be a risk factor for the manifestation or progression of BCRL (OR 0.94, *p* = 0.862). Two studies found that breast reconstruction did not have a significant impact on the incidence of BCRL regardless of axillary intervention and reconstruction type [37, 38].

#### Seroma

Two studies looked at the development of a seroma as a risk factor for the manifestation of progression of lymphedema [41, 42]. One study was a prospective cohort [41] and the other was case-controlled [42]. Ten participants had BCRL and 602 were at risk for BCRL. Conflicting evidence exists as Ribeiro-Pereira et al. found patients at risk for BCRL who developed a seroma demonstrated a higher incidence to lymphedema (HR 1.46, 95% CI 1.14–1.87, *p* = 0.003), while Shahpar et al. [42] found that the presence of a seroma did not demonstrate a significant correlation with the development of BCRL (OR 0.85, 95% CI 0.4–1.81, *p* = 0.679).

#### Trauma events

Eight studies looked at trauma events as potential risk factors for the manifestation or progression of BCRL [8, 11, 17, 35, 42–45]. The included studies consisted of one randomized controlled trial [44], four prospective cohort studies [8, 11, 17, 45], two case-controlled [35, 42], and one case study [43]. The trauma events ranged from less serious (e.g., bruises) to more serious (e.g., fractures). Twenty-two participants had BCRL, 242 were at risk for BCRL, and 885 were either at risk for or had BCRL. Six of the eight articles did not report a trauma event resulting in the manifestation or progression of BCRL [8, 11, 17, 35, 44, 45]. Conversely, Shahpar et al. [42] found trauma to be a risk factor in the development of BCRL (OR 2.71, 95% CI 1.16–6.33, *p* = 0.02) and Li et al. [43] found prolonged sun exposure to result in BCRL of the chest wall.

#### Heavy exercise

One retrospective cohort study looked at heavy exercise (> 80% one repetition maximum) as a risk factor for the manifestation of BCRL (*N* = 149) [46]. No associations were found between performing heavy resistance training and the development of BCRL.

#### Wound infection

One prospective cohort study examined the relationship between wound infection and lymphangitis with the development of BCRL [47]. In a sample of 455 participants with BCRL, more than half had reported a prior wound infection (OR 3.11, 95% CI 1.41–6.82, *p* = 0.003) and lymphangitis (OR 3.83, 95% CI 1.57–9.34, *p* = 0.002) in the ipsilateral arm.

### Evidence map of blood pressure as a risk factor

**Fig. 3:**
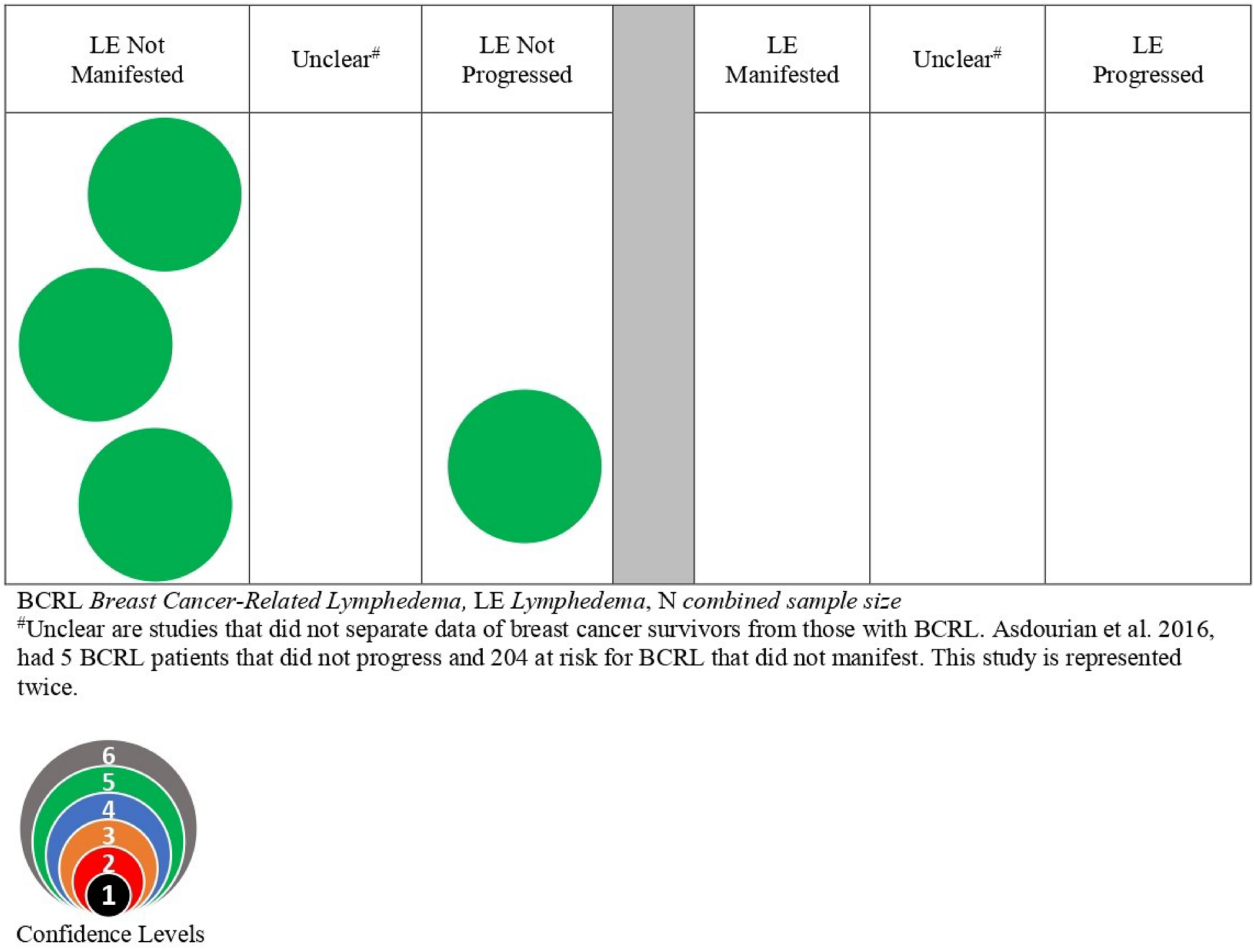
Precautionary risk of taking blood pressure on breast cancer survivors and BCRL

The results for blood pressure measurements to the ipsilateral limb as a risk factor for the manifestation or progression of BCRL are presented in the bubble map (Fig. 3) and Table 2 and include three studies [8, 11, 17]. All three studies were prospective cohort studies and included a total of 941 participants at risk for BCRL and 5 with BCRL. Having one or more blood pressure measurements to the ipsilateral limb was not associated with an increase in limb volume [8, 11, 17], even in participants who had undergone axillary lymph node dissection (ALND) [17].

**Table 2:** Blood pressure study characteristics and outcomes

Author (year)	Study type; level of evidence; confidence level	Sample size, diagnosis	Outcomes
Asdourian et al. (2016) [11]	Prospective cohort; Level 4; Level 5	*N* = 204 at risk for BCRL *N* = 5 with BCRL	Univariate analysis: having one or more blood pressure measurements versus none was significantly associated with decreased weight associated volume change (95% CI − 1.26 to 0.03, *p* = 0.0109); this was no longer significant upon multivariate analysis
Ferguson et al. (2016) [17]	Prospective cohort; Level 4; Level 5	*N* = 482 at risk for BCRL	Univariate analysis: no significant association between increased volume change and having blood pressure readings (*p* = 0.034) – In patients who underwent ALND, blood pressure (*p* = 0.39) was not associated with increased arm swelling – Patients with a BMI ≥ 25 lb/in^2^ at time of diagnosis (*p* = 0.0064), undergoing ALND (*p* = 0.0003), having blood pressure readings (*p* = 0.034), RLNR (*p* < 0.001), and cellulitis (*p* = 0.001) were significantly associated with arm volume increases
Kilbreath et al. (2016) [8]	Prospective cohort; Level 4; Level 5	*N* = 255 at risk for BCRL	Univariate analysis/ROC: ≥ 1 blood pressure measurement (OR 1.3, 95% CI 0.5–3.6, *p* = 0.6) was not retained as risk factor for lymphedema in the at-risk limb

*ALND* axillary lymph node dissection, *BCRL* breast cancer-related lymphedema, *BMI* body mass index, *CI* confidence interval, *OR* odds ratio, *RLNR* regional lymph node irradiation

### Evidence map of temperature as a risk factor

**Fig. 4:**
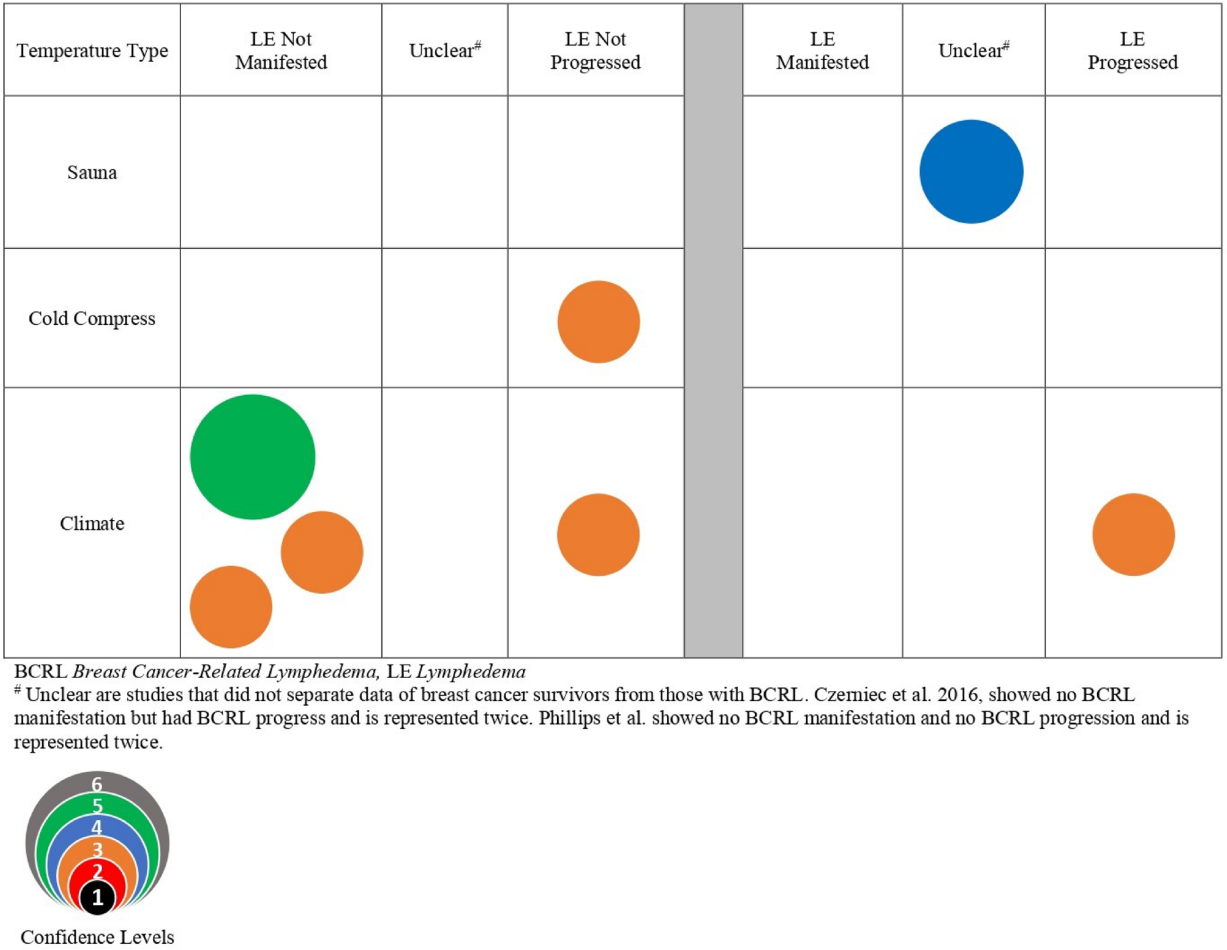
Precautionary risk of temperature on breast cancer survivors and BCRL

The results for the impact of temperature on the ipsilateral limb as a risk factor for the manifestation or progression of BCRL are presented in the bubble map (Fig. 4) and Table 3. Six articles relevant to temperature spanning cryotherapy, climate, hot tub use, and sauna use were included. The study types include one randomized controlled trial [48], four prospective cohort studies [8, 45, 49, 50], and one case-controlled [35]. Eighty-four participants had BCRL, 131 were at risk for BCRL, and 280 had BCRL or were at risk for BCRL. Askary et al. [48] found that pulsed local cryotherapy to the ipsilateral limb resulted in a decrease in thickness and circumferential limb difference at the wrist, below the elbow, and above the elbow after 6 weeks (*p* < 0.01) and 12 weeks (*p* < 0.001) of treatment compared to the control group. Three studies found that climate did not significantly impact limb volume in terms of manifestation of the progression of BCRL [8, 45, 50]. Conversely, Czerniec et al. [49] found a correlation between weather and arm volume where the maximum temperature on the day prior to limb volume measurements affected extracellular fluid (*r* = 0.27, *p* < 0.001), arm volume (*r* = 0.23, *p* < 0.001), and self-reported swelling (*r* = 0.26, *p* < 0.001). Two studies found hot tub use was not a significant risk factor in the manifestation or progression of BCRL [35, 45]. Conflicting findings exist regarding sauna use as a risk factor. One study did not identify it as a risk factor [35]; however, Showalter et al. [45] found it to be a risk factor in both univariate (OR 5.77, 95% CI 1.00–33.82, *p* = 0.05) and multivariate analysis (OR 6.67, 95% CI 1.36–32.56, *p* = 0.01). Additionally, Showalter et al. [45] found a significant interaction also existed between sauna use and having a cut on the ipsilateral limb associated with arm swelling (OR 18.74, 95% CI 1.41–294.48, *p* = 0.027).

**Table 3:** Temperature study characteristics and outcomes

Author (year)	Study type; level of evidence; confidence level	Sample size, diagnosis	Independent variable	Outcomes
Askary and Elshazly (2022) [48]	Randomized controlled trial; Level 3; Level 3	*N* = 40 with BCRL	Pulsed local cryotherapy	Multivariate analysis: in patients who received cryotherapy, there was a significant decrease in thickness and circumferential limb difference at the wrist, below the elbow, and above the elbow after 6 weeks (*p* < 0.01) and 12 weeks (*p* < 0.001) of treatment compared to the control group
Czerniec et al. (2016) [49]	Case control; Level 2; Level 3	*N* = 26 with BCRL	Climate	Coefficients of variations: arm volume and ECF did not vary significantly (2.3% and 3.7%, respectively) Pearson correlation: correlation of weather and lymphedema demonstrates that temperature had an effect on BCRL, with the max temperature on the day before measurement slightly affecting ECF (*r* = 0.27, *p* < 0.001), arm volume (*r* = 0.23, *p* < 0.001), and self-reported swelling (*r* = 0.26, *p* < 0.001)
Kilbreath et al. (2016) [8]	Prospective cohort; Level 4; Level 5	*N* = 112 at risk for BCRL (*N* = 47 with ≥ 3 episodes; *N* = 65 with ≤ 3 episodes)	Climate	Univariate analysis/ROC: extreme heat (OR 0.6, 95% CI 0.2–1.7, *p* = 0.37) was not retained as risk factor for lymphedema in the at-risk limb
Phillips et al. (2023) [50]	Prospective cohort; Level 3; Level 3	*N* = 14 with BCRL *N* = 11 at risk for BCRL	Climate	Repeated measures analysis of covariance: no significant difference was found in limb circumference (*p* = 0.48; *p* = 0.72), limb volume (*p* = 0.94; *p* = 0.97), or bioimpedance (*p* = 0.89; *p* = 0.18) measures across the timepoints in participants at risk for and diagnosed with BCRL, respectively
Showalter et al. (2013) [45]	Prospective cohort; Level 3; Level 4	Exercise in hot weather: *N* = 54 Travel to hot/humid place: *N* = 138 Hot tub use: *N* = 75 Sauna use: *N* = 13 (All at risk of or with BCRL)	Climate, hot tub use, sauna use	Univariate analysis: over a 12-month period of time, exercise in hot weather (OR 1.00, 95% CI 0.11–4.17, *p* = 0.99), travel to hot/humid places (OR 1.09, 95% CI 0.40–2.96, *p* = 0.87), and hot tub use (OR 0.76, 95% CI 0.17–3.31, *p* = 0.71) were not significantly associated with incident arm swelling, but sauna use was (OR 5.77, 95% CI 1.00–33.82, *p* = 0.05) Multivariate analysis: sauna use remained significantly associated with incident arm selling (OR 6.67, 95% CI 1.36–32.56, *p* = 0.01); a significant interaction also existed between sauna use and having a cut on the ipsilateral limb associated with arm swelling (OR 18.74, 95% CI 1.41–294.48, *p* = 0.027)
Swenson et al. (2009) [35]	Case–control; Level 2; Level 4	*N* = 4 with BCRL *N* = 8 at risk for BCRL	Whirlpool, hot tub, or sauna use	Univariate analysis: whirlpool, hot tub, or sauna use was not a significant variable in the development of or exacerbation of lymphedema (OR 0.5, *p* = 0.258)

*BCRL* breast cancer-related lymphedema, *CI* confidence interval, *ECG* extracellular fluid, *OR* odds ratio

### Evidence map of air travel as a risk factor

**Fig. 5:**
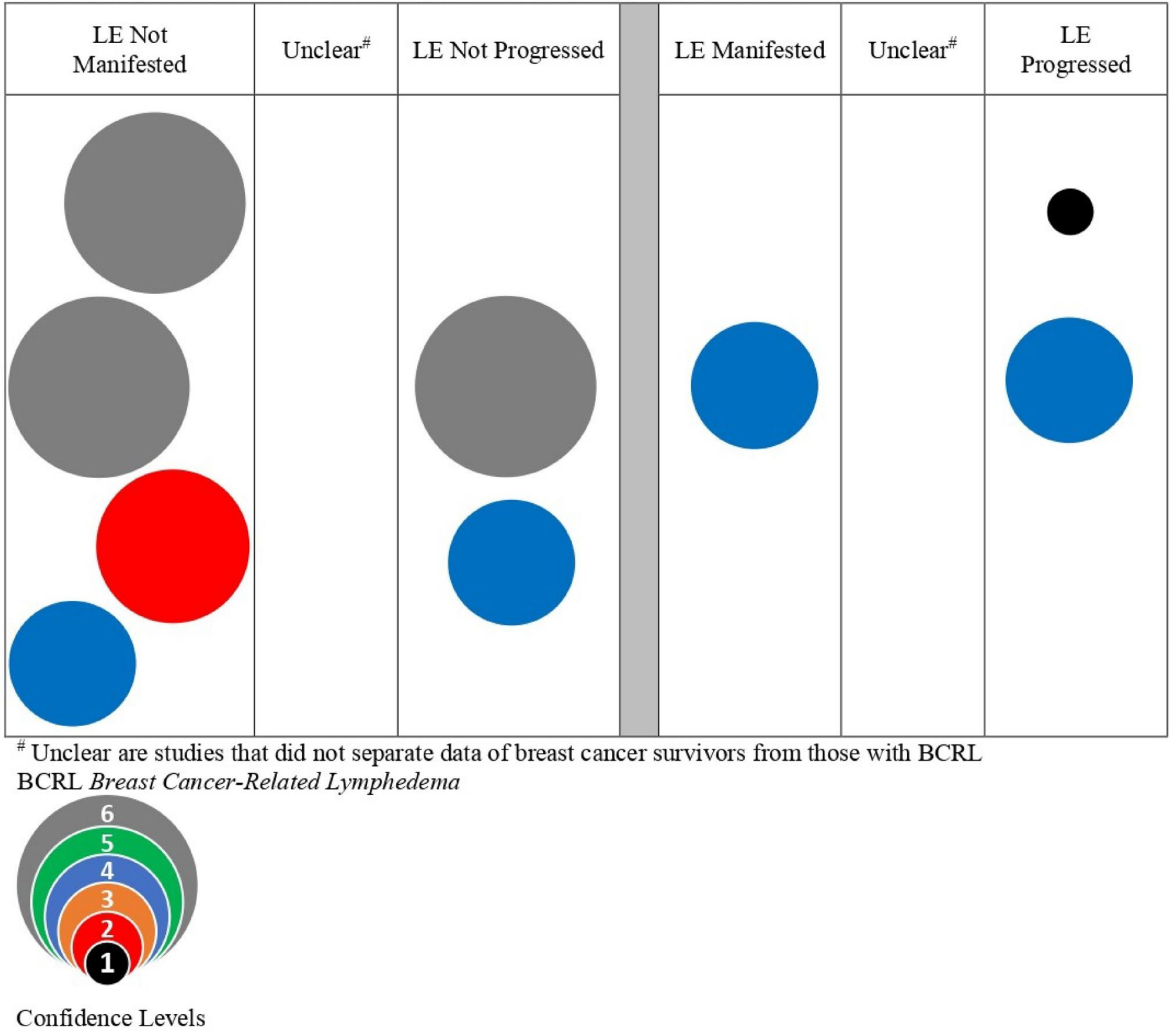
Precautionary risk of air travel on breast cancer survivors and BCRL

The results for air travel as a risk factor for the manifestation or progression of BCRL are presented in the bubble map (Fig. 5) and Table 4 and include six articles. The included articles consist of three prospective cohort studies [8, 17, 51], one cross-sectional [22], one case–control [35], and one case study [52]. Two hundred and two participants had BCRL and 1686 were at risk for BCRL. For the majority of participants, air travel, long-haul, and short-haul flights did not demonstrate a significant association with increased limb volume [8, 17, 22, 35, 51]. While not significant, Kilbreath et al. [22] noted that six participants experienced an inter-limb impedance ratio increase of ≥ 5% after air travel indicating a worsening of lymphedema (*N* = 2) and manifestation of lymphedema (*N* = 4). Similarly, Ward et al. [52] highlight fluctuations in inter-limb impedance ratios in a case study of a patient who flew on 20 separate occasions where the ratio increased and worsened after flying (Fig. 5).

**Table 4:** Air travel study characteristics and outcomes

Author (year)	Study type; level of evidence; confidence level	Sample size, diagnosis	Outcomes
Ferguson et al. (2016) [17]	Prospective cohort; Level 4; Level 6	*N* = 878 at risk for BCRL	Univariate analysis: no significant association between increased volume change and undergoing number of flights [(one or two, *p* = 0.77) (3 or more, *p* = 0.91) versus none)], or duration of flight [(1–12 h, *p* = 0.43) and (12 or more hours, *p* = 0.54) versus none)] – The number (*p* = 0.34) or duration of flights (*p* = 0.98) was not associated with an increase in limb volume change
Kilbreath et al. (2010) [22]	Cross-sectional; Level 3; Level 4	*N* = 72 at risk for BCRL	For 95% of participants, air travel did not adversely affect the inter-limb impedance ratio. Data suggest there is small risk of the development of lymphedema as a consequence of long-haul air travel For long-haul travelers, impedance ratios were 1.007 ± 0.064 prior to the flight and 1.006 ± 0.087 following the flight For short-haul travelers, impedance ratios were 0.994 ± 0.033 prior to the flight and 1.001 ± 0.038 following the flight
Kilbreath et al. (2016) [8]	Prospective cohort; Level 4; Level 5	*N* = 211 at risk for BCRL	Univariate analysis/ROC: International (OR 1.5, 95% CI 0.5–4.1, *p* = 0.45) and domestic (OR 0.9, 95% CI 0.4–3.0, *p* = 0.79) air travel were not retained as risk factors for lymphedema in the at-risk limb
Koelmeyer et al. (2022) [51]	Prospective cohort; Level 3; Level 6	*N* = 456 at risk for BCRL *N* = 156 with BCRL	No statistically significant association of any air travel (*p* = 0.365) was observed with the development or progression of BCRL
Swenson et al. (2009) [35]	Case–control; Level 2; Level 4	*N* = 69 at risk for BCRL *N* = 46 with BCRL	Univariate analysis: air travel was not a significant variable in the development of or exacerbation of lymphedema (OR 0.23, *p* < 0.001) Multivariate analysis: air travel was not a significant variable in the development of or exacerbation of lymphedema (OR 0.31, 95% CI 0.08–1.22, *p* = 0.093)
Ward et al. (2009) [52]	Case study; Level 1; Level 1	*N* = 1 with BCRL	Inter-arm impedance ratios fluctuated over time, generally increasing, and worsening following air travel. While there is no clear relationship between lymphedema status and the duration of flying time, these results could indicate a relationship with progression of lymphedema with air travel

*BCRL* breast cancer-related lymphedema, *CI* confidence interval, *OR* odds ratio

### Evidence map of behavior change as a risk factor

**Fig. 6:**
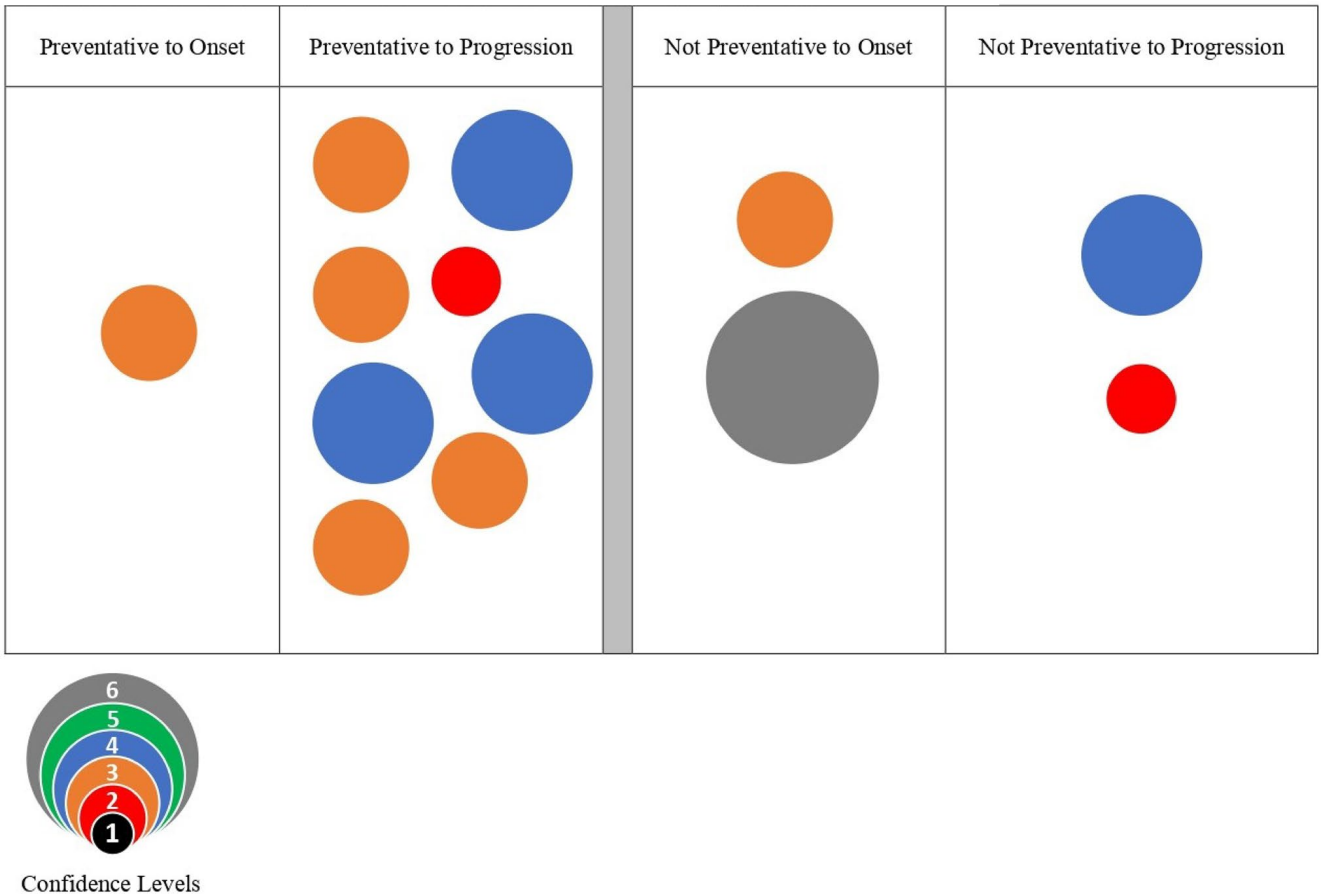
Behavioral interventions for preventing lymphedema onset and progression

The results for behavior change as a risk factor for the manifestation or progression of BCRL are presented in the bubble map (Fig. 6) and Table 5 and include 13 articles. The included articles consist of six randomized controlled trials [53–58], three prospective cohort studies [6, 59, 60], two quasi-experimental studies [61, 62], one cross-sectional study [63], and one case–control study [64]. Five hundred and seventy-three participants had BCRL and 765 were at risk for BCRL. Behavior change interventions varied across the studies including a variety of interventions often used in combination with each other including exercise, deep breathing, self-manual lymphatic drainage, self-monitoring of symptoms, use of compression, education (face-to-face and/or written), and skin care. Conflicting evidence exists as to whether participation in and/or adherence to a behavior change intervention prevented or improved BCRL in the included participants. Four articles did not demonstrate significant differences in arm volume or bioimpedance scores with participation and/or adherence to a behavior change intervention in participants with BCRL [6, 53, 55, 63]. Conversely, four articles found significant improvements over time in limb volume with participation in a self-management program for those with BCRL [54, 56, 61, 64]. While Liu et al. [62] noted that a majority of their participants with BCRL demonstrated limb volume improvements over time with behavior change, a small percentage (5%; 2/40) progressed from subclinical to mild lymphedema. Ochalek et al. [60] reported most of their participants with BCRL were able to maintain volume reduction with self-management 5-years post-intensive Complete Decongestive Therapy; however, those who were not adherent with self-management demonstrated an average 14% volume increase at the 5-year timepoint.

**Table 5:** Behavior change study characteristics and outcomes

Author (year)	Study type; level of evidence; confidence level	Sample size, diagnosis	Independent variable	Outcomes
Arinaga et al. (2019) [53]	Randomized controlled trial; Level 3; Level 4	*N* = 43 with BCRL	Self-care program: exercise, deep breathing, lymphatic drainage, skin care	Friedman test: no significant difference in L-Dex scores between the control group (*p* = 0.559) and intervention group (*p* = 0.71)
Brown et al. (2015) [6]	Prospective cohort; Level 3; Level 3	*N* = 118 with BCRL *N* = 10 at risk for BCRL	Adherence to BCRL modalities: self-monitoring of heaviness, tightness, swelling; circumferential difference; long-term wear of compression garments, CDT; skin care; exercise; body weight	Linear regression: adherence to self-care activities did not predict experiencing BCRL outcomes at 12 months Multivariable-adjusted sensitivity analysis: adherence to BCRL self-care activities did not predict changes in volumetry (< 25% compared with ≥ 75% of adherence, *β* = 0.09 and *p*_trend_ = 0.741), sum of arm circumferences (< 25% compared with ≥ 75% of adherence, *β* = − 0.63 and *p*_trend_ = 0.693), bioimpedance spectroscopy (< 25% compared with ≥ 75% of adherence, *β* = 4.04 and *p*_trend_ = 0.753), or lymphedema symptoms (< 25% compared with ≥ 75% of adherence, *β* = − 0.14 and *p*_trend_ = 0.306)
Cansız et al. (2022) [61]	Quasi-experimental; Level 3; Level 3	*N* = 44 with BCRL	Self-management lymphedema education program: face-to-face sessions and information booklet including skin care, compression therapy, simple lymphatic drainage, exercise	Wilcoxon test: metacarpal phalangeal circumference (*p* = 0.018, ES = − 0.36) and volumetric differences (*p* = 0.0000, ES = − 0.79) showed a significant change over time
Du et al. (2022) [54]	Randomized controlled trial; Level 4; Level 4	*N* = 92 with BCRL	*TOLF* program: teaches self-management strategies to activate lymphatic system and promote lymph flow	Mann–Whitney *U* test: at the study endpoint (3 months), the TOLF group had significantly fewer patients with ≥ 5% arm volume differences compared to -control group ([5 out of 45] vs [13 out of 43], *p* = 0.035) – 12.8% of those in TOLF group saw reduction in ≥ 5% arm volume differences from baseline to post-intervention compared to the control group which saw a 1.9% increase in the proportion of patients with ≥ 5% arm volume differences
Fu et al. (2014) [59]	Prospective, longitudinal, quasi-experimental design with repeated measures; Level 3; Level 3	*N* = 140 at risk for BCRL	*TOLF* program: teaches self-management strategies to activate lymphatic system and promote lymph flow	Descriptive statistics: 97% of the patients maintained and improved their pre-operative limb volume. 4 patients developed measurable lymphedema. At the 12-month follow-up: among the 4 patients with measurable lymphedema, 2 patients' limb volume returned to the pre-operative level through continued participating in *TOLF*
Gençay Can et al. (2019) [64]	Case-controlled; Level 2; Level 2	*N* = 25 with BCRL (subclinical)	Education on lymphedema and risk factors, skin care advice, home-based exercise program	*t* test/Wilcoxon signed-rank test: volume of the affected arm (*p* = 0.01) and percentage volume difference between the arms (*p* < 0.001) improved significantly at the end of the treatment (*p* < 0.05)
Imamoğlu et al. (2016) [63]	Cross-sectional, prospective; Level 2; Level 2	*N* = 38 with BCRL	Education on causes and symptoms of lymphedema, methods for minimizing complications from lymphedema, skin care, exercise, protective clothing	Descriptive statistics/chi-square test: no significant difference between the intervention and control groups in severity of lymphedema post-intervention *p* = 0.879
Jeffs and Wiseman (2013) [55]	Randomized controlled trial’ Level 4; Level 4	*N* = 23 with BCRL	Exercise program: series of gravity-resistive isotonic arm exercises in a sequence similar to MLD; reinforcement of self-care	Wilcoxon signed-rank test: statistically significant improvement in extra limb volume in the control group at week 26 (median = − 11.69%, 95% CI − 26.57 to − 5.12, *z* = − 2.50, *p* = 0.013, *r* = 0.79) compared to no effect in control group (median = − 9.2%, 95% CI − 17.71 to 1.1, *z* = − 1.64, *p* = 0.10, *r* = 0.28) Mann–Whitney *U* test: no statistically significant difference in % extra limb volume between the two groups at week 26 (*U* = 40.0, *z* = − 1.319, *p* = 0.187, *r* = 0.28)
Ligabue et al. (2019) [56]	Randomized controlled trial; Level 4; Level 4	*N* = 41 with BCRL	Self-administered CDT: self-MLD, self-bandage, breathing, mobilization exercises, education	Mann–Whitney *U* test: asymmetry between the affected and unaffected arms and hands decreased in the experimental group at 1 month (median reduction: 5%, *p* = 0.015; median reduction 3%, *p* = 0.030) and 6 months (median reduction: 8%, *p* = 0.001; median reduction: 8%, *p* = 0.015), respectively
Liu et al. (2021) [62]	Quasi-experimental; Level 3; Level 3	*N* = 41 with BCRL	*TOLF* program: teaches self-management strategies to activate lymphatic system and promote lymph flow	Descriptive statistics: limb circumference difference was decreased at 1-month follow-up and was maintained at 12-months – *β* values of limb circumference difference at each follow-up point were − 0.205 (*p* < 0.001), − 0.188 (*p* < 0.001), − 0.176 (*p* = 0.01), and − 0.155 (*p* = 0.006) indicating that the limb circumference difference was significantly lower at 4 follow-ups compared with baseline – Among those who finished the last follow-up, 77.5% (31/40) maintained their baseline lymphedema status, 17.5% (7/40) reversed the lymphedema status from mild to subclinical, and 5% (2/40) progressed from subclinical to mild lymphedema
Ochalek et al. (2015) [60]	Prospective cohort; level 3; Level 3	*N* = 60 with BCRL	Maintenance phase of CDT: compression garment use, skin care, exercise, follow-up appointments	Descriptive statistics: all participants had undergone intensive CDT and achieved significant volume reduction [278.2 mL (13.1%) and 283.7 mL (11.1%)] – After 5-years of self-management, those adherent with maintenance CDT maintained volume reductions, while those not adherent had an average volume increase of 399.2 mL (14%)
Paskett et al. (2021) [57]	Randomized controlled trial; Level 4; Level 6	*N* = 554 at risk for BCRL	LEAP program: education, compression garments, exercise	Descriptive statistics: at 18 months, lymphedema incidence did not differ between groups as 58% (141) of the control group (education only) and 55% (172) (*p* = 0.37) of the LEAP group were free of lymphedema
Temur et al. (2019) [58]	Randomized controlled trial; Level 3; Level 3	*N* = 61 at risk for BCRL	Self-management program: training booklet describing exercise, MLD, and prevention methods; regular follow-up	Mann–Whitney *U* test: in the first, third, and sixth months, limb measurements in the intervention group were significantly lower than the control group (*p* < 0.05) Kruskal–Wallis *H* Test: differences between the intervention and control groups for the development of lymphedema were statistically significant (*χ*^2^ = 25.943; *p* = 0.000)

*BCRL* breast cancer-related lymphedema, *CDT* complete decongestive therapy, *CI* confidence interval, *EF* effect size, *LEAP* lymphedema education and prevention, *MLD* manual lymphatic drainage, *Md* median, *TOLF* the-optimal-lymph-flow

Patients at risk for BCRL either maintained or improved their pre-operative limb volume by participating in a self-management program [58, 59], whereas Paskett et al. [57] did not find a difference in limb volume in those who participated in self-care compared to controls.

## Discussion

The trajectory of survival post-breast cancer treatment has seen a dramatic swing over the past several decades secondary to advances in the medical management of the disease. However, survivorship carries the burden of long-term sequelae including BCRL. Given the insidious nature of this condition, proactive and persistent surveillance is crucial in limiting the manifestation and/or progression of the disease. For decades, risk reduction practices have existed to guide those at risk and diagnosed with BCRL; however, many of these practices are anecdotal in nature and have been more recently challenged in the literature. The purpose of this study was to (1) review if current evidence supports or refutes patient precautions for the purpose of preventing the manifestation and/or progression of BCRL, (2) review if behavioral changes result in the prevention or reduction of BCRL, and (3) identify related gaps of knowledge for future research.

### Trauma

Trauma is broad category ranging from cuts and scratches to surgery. Evidence is categorized to explore the relative risk of trauma events for BCRL. Overall, evidence for ipsilateral blood draws and medical skin punctures as risks for BCRL trended toward refuting the precaution. The evidence for ipsilateral surgery as a risk factor for BCRL was mixed, while seroma and infection were retained as risk factors for BCRL.

Blood draws in the ipsilateral upper extremity were not a risk factor for BCRL [8, 11, 17]. Similar findings were reported in a recent systematic review which found limited evidence for blood draws increasing the risk of BCRL [65].

The evidence related to medical skin puncture was not generally associated with increased risk for BCRL in prospective studies. Two smaller studies [34, 35] did, however, associate increased risk for BCRL with skin puncture, with one study [35] focused on the association between multiple fluid aspirations and risk of BCRL. A recent systematic review of the effects of skin puncture on the risk of BCRL affirmed our finding that skin puncture should not be retained as a risk factor for BCRL [66].

Evidence for surgery in the ipsilateral upper quadrant as a risk factor for BCRL was mixed, with three studies reporting manifestation or temporary exacerbation of BCRL [36, 39, 40] and three studies indicating surgery is not a risk factor for BCRL [35, 37, 38]. While three studies found risk associated with surgery, two of those studies reported the resolution of volume increases at follow-up ranging from 2-weeks [40] to 33 months [39].

Seroma was retained as a risk factor because it was related to an increased risk for BCRL [41], although Shahpar et al. [42] did not associate seroma with the manifestation or progression of BCRL. The findings of a meta-analysis by Shen et al. [2] similarly found that patients with post-operative outcomes that included seroma were 25.3% more likely than those without seroma to develop BCRL. Wound infection was also retained as a risk for BCRL. Recent studies reported that wounds were associated with BCRL risk increases of 10% [67] to 80.3% [2].

### Blood pressure

In several prospective studies, blood pressure measurement in isolation did not appear to be a risk factor for BCRL [8, 11, 17]. A recent review similarly found no increased risk for BCRL with ipsilateral blood pressure measurement [2].

### Temperature

Temperature was generally not associated with increased risk for BCRL; however, there was limited evidence that sauna use [45] and climate temperature [49] could increase the risk for BCRL.

### Air travel

Air travel did not seem to be a risk factor for BCRL [8, 17, 22, 35, 51], although two studies [22, 52] reported inter-limb impedance ratio increases in several individuals which did not reach significance. A recent prospective surveillance study had similar findings that air travel was not a risk factor for BCRL [51].

### Behavioral change

Evidence for lymphedema prevention through behavioral change is mixed. However, 9 of the 13 included studies in this review evidenced that behavioral change reduced the risk of BCRL. As found by Perdomo et al. [68] in a systematic review, lymphedema prevention education interventions were diverse, and varied in time and mode of delivery. Studies infrequently assess knowledge, making the efficacy of education programs difficult to assess.

### Evidence gaps

Gaps persist in high-quality evidence related to reducing BCRL risk. Although evidence about BCRL risk factors (trauma, blood pressure measurement, temperature, and air travel) and programs to minimize risk of BCRL have increased, there is an interesting division between study populations. Most participants in risk factor studies have been women at risk for BCRL. Risks for BCRL progression in women who already have BCRL have been studied less frequently. Conversely, behavioral change programs for reducing risk factors for BCRL have primarily been trialed with women who already have BCRL rather than including more women at risk. These gaps in evidence about BCRL progression with risk factor exposure and the efficacy of behavioral change programs for reducing the risk of BCRL manifestation in those at risk require additional study.

Evidence gaps are also attributable to study methods. Many studies were eliminated from this review because they lacked objective lymphedema measurements. Self-report measures are limited in their efficacy for prognostic studies. The inclusion of objective limb and trunk measurements and demographic data (e.g., body mass index and race/ethnicity) would increase rigor and add to the specificity of findings. Rigorous prospective studies are needed to fill these evidence gaps.

## Strengths and limitations

This mapping review used a multi-database search and systematic review citation screening to ensure that relevant literature was not disregarded. Our extensive inclusion criteria strengthened our finding that there are some gaps in the literature on the topic of risk factors that contribute to the manifestation and progression of BCRL. While it is not possible to translate the findings to all settings and populations, the inclusion criteria facilitated studies that had populations outside of high-volume research and/or medical institutions. The largest confidence levels were attributed to prospective cohort studies. Compared to retrospective observational study designs, prospective designs align more closely with causal inferences. However, it is worth noting that there are limitations to prospective designs that prevent them from proving causal relationships. These include the possibility of unmeasured confounding variables, selection bias, and attrition. Therefore, the studies in this review with the highest confidence levels have inferred relationships but caution should be employed when confirming causality, especially for the chronic disease of BCRL that has variability in the latency of both manifestation and progression. With the results of other study designs, this review gives a more robust summary of risk factor causal evidence of BCRL and the knowledge gap.

This mapping review presents with random error due to the heterogeneity of the study designs and associated variability in the methodology of the included studies. In addition, the review included only English-language publications, which may have limited the data. Study populations, short follow-up periods, few end-points, and vagueness separating the manifestation and progression of lymphedema in some included studies may have influenced the interpretation of the knowledge gap and risk factor causality. The extensive list of factors that could be categorized as trauma, thermal exposure, and insult to the skin were not separated to properly analyze each risk; however, should an extensive analysis be conducted the effect sizes would likely be marginal. Considering the chronicity of BCRL, the included studies were limited in assessing the risk factors as they relate to other comorbidities of this disease. This includes the development of fibrosis which is considered a component of BCRL classification by the International Society of Lymphology. There is insufficient long-term data about the incidence of BCRL; however, cases of BCRL appear to lessen after 5 years post-lymphadenectomy and /or irradiation [69]. Nonetheless, there are known cases of BCRL that develop after this 5-year timeframe, and this cohort is underrepresented in the reviewed studies. It is also important to recognize that the manifestation and/or progression of BCRL observed in the included studies may have been provoked by other pathologic comorbidities that were not measured or informed by the participants. In a similar vein, the absence of manifestation and/or progress may have been due to undisclosed self-administered precautions and interventions.

## Conclusion

Within the confines of limb and trunk circumferential and/or volume enlargement, a ‘just in case’ approach to patient education on risk factors may not be appropriate for breast cancer survivors at risk of manifesting lymphedema. Evidence is sufficient to refute many of the previously claimed risk factors for the manifestation of BCRL. However, there was scant evidence about lymphedema progression, limiting the interpretation for this cohort at risk for the progression of BCRL, which highlights the need for further study. There is evidence that suggests behavioral change influences a reduced risk of progressing BCRL; however, the same cannot be stated about the influence on behavioral change and the manifestation of BCRL. Patient education about precautionary risks needs to be concordant with what is currently evidenced in the literature.

Future studies should continue to focus on prospective cohort designs but should consider extending past 5 years and include non-volume-related outcome measures (e.g., fibrosis, pain, sensation, functional measures, and quality of life) associated with the burden of this chronic disease. This may require an approach that involves data collection in rehabilitation and primary care settings.

## Data Availability

Not applicable.

## References

[1] Key Statistic for Breast Cancer. American Cancer Society. 2023. https://www.cancer.org/cancer/types/breast-cancer/about/how-common-is-breast-cancer.html. Accessed 30 June 2023.

[2] Shen A, Lu Q, Fu X, et al. Risk factors of unilateral breast cancer-related lymphedema: an updated systematic review and meta-analysis of 84 cohort studies. Support Care Cancer. 2022;31(1):18. 10.1007/s00520-022-07508-2. 36513801 10.1007/s00520-022-07508-2

[3] McLaughlin SA, Brunelle CL, Taghian A. Breast cancer-related lymphedema: risk factors, screening, management, and the impact of locoregional treatment. J Clin Oncol. 2020;38(20):2341. 32442064 10.1200/JCO.19.02896 PMC7343436

[4] Pappalardo M, Starnoni M, Franceschini G, Baccarani A, De Santis G. Breast cancer-related lymphedema: recent updates on diagnosis, severity and available treatments. J Pers Med. 2021;11(5):402. 34065795 10.3390/jpm11050402 PMC8151072

[5] Anderson EA, Armer JM. Factors impacting management of breast cancer-related lymphedema (BCRL) in Hispanic/Latina breast cancer survivors: a literature review. Hispanic Health Care Int. 2021;19(3):190–202. 10.1177/1540415321990621 PMC8353654 33550878

[6] Brown JC, Kumar A, Cheville AL, et al. Association between lymphedema self-care adherence and lymphedema outcomes among women with breast cancer-related lymphedema. Am J Phys Med Rehabil. 2015;94(4):288–96. 10.1097/phm.0000000000000178. 25171662 10.1097/PHM.0000000000000178 PMC4344918

[7] Rafn BS, Christensen J, Larsen A, Bloomquist K. Prospective surveillance for breast cancer-related arm lymphedema: a systematic review and meta-analysis. J Clin Oncol. 2022;40(9):1009–26. 35077194 10.1200/JCO.21.01681

[8] Kilbreath SL, Refshauge KM, Beith JM, et al. Risk factors for lymphoedema in women with breast cancer: a large prospective cohort. Breast. 2016;28:29–36. 10.1016/j.breast.2016.04.011. 27183497 10.1016/j.breast.2016.04.011

[9] Shaitelman SF, Chiang YJ, Griffin KD, et al. Radiation therapy targets and the risk of breast cancer-related lymphedema: a systematic review and network meta-analysis. Breast Cancer Res Treat. 2017;162(2):201–15. 10.1007/s10549-016-4089-0. 28012086 10.1007/s10549-016-4089-0

[10] Warren LE, Miller CL, Horick N, et al. The impact of radiation therapy on the risk of lymphedema after treatment for breast cancer: a prospective cohort study. Int J Radiat Oncol Biol Phys. 2014;88(3):565–71. 10.1016/j.ijrobp.2013.11.232. 24411624 10.1016/j.ijrobp.2013.11.232 PMC3928974

[11] Asdourian MS, Skolny MN, Brunelle C, Seward CE, Salama L, Taghian AG. Precautions for breast cancer-related lymphoedema: risk from air travel, ipsilateral arm blood pressure measurements, skin puncture, extreme temperatures, and cellulitis. Lancet Oncol. 2016;17(9):e392-405. 10.1016/s1470-2045(16)30204-2. 27599144 10.1016/S1470-2045(16)30204-2

[12] Tendero-Ruiz L, Palomo-Carrión R, Megía-García-Carpintero Á, Pérez-Nombela S, López-Muñoz P, Bravo-Esteban E. The effect of therapeutic exercise in the prevention of lymphoedema secondary to breast cancer: a systematic review. Arch Med Sci. 2023;19(6):1684–92. 10.5114/aoms.2020.101435. 38058721 10.5114/aoms.2020.101435 PMC10696974

[13] Panchik D, Masco S, Zinnikas P, et al. Effect of exercise on breast cancer-related lymphedema: what the lymphatic surgeon needs to know. J Reconstr Microsurg. 2019;35(1):37–45. 10.1055/s-0038-1660832. 29935493 10.1055/s-0038-1660832

[14] McEvoy MP, Gomberawalla A, Smith M, et al. The prevention and treatment of breast cancer-related lymphedema: a review. Front Oncol. 2022;12:1062472. 10.3389/fonc.2022.1062472. 36561522 10.3389/fonc.2022.1062472 PMC9763870

[15] Jammallo LS, Miller CL, Singer M, et al. Impact of body mass index and weight fluctuation on lymphedema risk in patients treated for breast cancer. Breast Cancer Res Treat. 2013;142(1):59–67. 10.1007/s10549-013-2715-7. 24122390 10.1007/s10549-013-2715-7 PMC3873728

[16] Sherman KA, Koelmeyer L. Psychosocial predictors of adherence to lymphedema risk minimization guidelines among women with breast cancer. Psychooncology. 2013;22(5):1120–6. 10.1002/pon.3111. 22689156 10.1002/pon.3111

[17] Ferguson CM, Swaroop MN, Horick N, et al. Impact of ipsilateral blood draws, injections, blood pressure measurements, and air travel on the risk of lymphedema for patients treated for breast cancer. J Clin Oncol. 2016;34(7):691–8. 10.1200/jco.2015.61.5948. 26644530 10.1200/JCO.2015.61.5948 PMC4872021

[18] National Lymphedema Network. Risk reduction practices. National Lymphedema Network. https://lymphnet.org/risk-reduction-practices. Accessed 14 Dec 2023.

[19] American Cancer Society. For people at risk of lymphedema. American Cancer Society. https://www.cancer.org/cancer/managing-cancer/side-effects/swelling/lymphedema/for-people-at-risk-of-lymphedema.html. Accessed 14 Dec 2023.

[20] National Cancer Institute. Lymphedema and cancer treatment. National Cancer Institute. https://www.cancer.gov/about-cancer/treatment/side-effects/lymphedema. Accessed 14 Dec 2023.

[21] Susan G. Komen. Understanding lymphedema—Komen perspectives. Susan G. Komen Organization. https://www.komen.org/blog/komen-perspectives-understanding-lymphedema/. Accessed 14 Dec 2023.

[22] Kilbreath SL, Ward LC, Lane K, et al. Effect of air travel on lymphedema risk in women with history of breast cancer. Breast Cancer Res Treat. 2010;120(3):649–54. 10.1007/s10549-010-0793-3. 20180016 10.1007/s10549-010-0793-3

[23] Cemal Y, Pusic A, Mehrara BJ. Preventative measures for lymphedema: separating fact from fiction. J Am Coll Surg. 2011;213(4):543–51. 10.1016/j.jamcollsurg.2011.07.001. 21802319 10.1016/j.jamcollsurg.2011.07.001 PMC3652571

[24] Gandhi A, Xu T, Desnyder SM, et al. Prospective, early longitudinal assessment of lymphedema-related quality of life among patients with locally advanced breast cancer: the foundation for building a patient-centered screening program. The Breast. 2023;68:205–15. 10.1016/j.breast.2023.02.011. 36863241 10.1016/j.breast.2023.02.011 PMC9996356

[25] Jammallo LS, Miller CL, Horick NK, et al. Factors associated with fear of lymphedema after treatment for breast cancer. Oncol Nurs Forum. 2014;41(5):473–83. 10.1188/14.Onf.473-483. 25158653 10.1188/14.ONF.473-483

[26] Uhlmann RA, Mott SL, Curry M, et al. Analysis of the understanding and worry about lymphedema of patients with breast cancer. Ann Surg Oncol. 2022;29(10):6428–37. 10.1245/s10434-022-12189-6. 35913669 10.1245/s10434-022-12189-6

[27] Alcorso J, Sherman KA, Koelmeyer L, Mackie H, Boyages J. Psychosocial factors associated with adherence for self-management behaviors in women with breast cancer-related lymphedema. Support Care Cancer. 2016;24(1):139–46. 10.1007/s00520-015-2766-x. 25957012 10.1007/s00520-015-2766-x

[28] Miake-Lye IM, Hempel S, Shanman R, Shekelle PG. What is an evidence map? A systematic review of published evidence maps and their definitions, methods, and products. Syst Rev. 2016;5:28. 10.1186/s13643-016-0204-x. 26864942 10.1186/s13643-016-0204-x PMC4750281

[29] Ouzzani M, Hammady H, Fedorowicz Z, Elmagarmid A. Rayyan—a web and mobile app for systematic reviews. Syst Rev. 2016;5:1–10. 27919275 10.1186/s13643-016-0384-4 PMC5139140

[30] APTA clinical practice guideline process manual, revised. American Physical Therapy Association. 2023. https://www.apta.org/patient-care/evidence-based-practice-resources/clinical-practice-guidelines/cpg-development/cpg-development-manual. Accessed 1 Apr 2024.

[31] Kondo K, Low A, Everson T, et al. Health disparities in veterans: a map of the evidence. Med Care. 2017;55:S9–15. 28806361 10.1097/MLR.0000000000000756

[32] Hempel S, Taylor SL, Solloway MR, et al. Evidence map of acupuncture for pain. Evidence Map of Acupuncture [Internet]. Department of Veterans Affairs (US); 2014. 24575449

[33] Alem M, Gurgel MS. Acupuncture in the rehabilitation of women after breast cancer surgery—a case series. Acupunct Med. 2008;26(2):87–93. 18591908

[34] Clark B, Sitzia J, Harlow W. Incidence and risk of arm oedema following treatment for breast cancer: a three-year follow-up study. QJM. 2005;98(5):343–8. 15820971 10.1093/qjmed/hci053

[35] Swenson KK, Nissen MJ, Leach JW, Post-White J. Case-control study to evaluate predictors of lymphedema after breast cancer surgery. Oncol Nurs Forum. 2009;36(2):185–93. 10.1188/09.Onf.185-193. 19273407 10.1188/09.ONF.185-193

[36] Baltzer HL, Harvey J, Fox PM, Moran SL. De novo upper extremity lymphedema after elective hand surgery in breast cancer survivors. Ann Plast Surg. 2017;79(1):24–7. 10.1097/sap.0000000000000986. 28187025 10.1097/SAP.0000000000000986

[37] Basta MN, Fischer JP, Kanchwala SK, et al. A propensity-matched analysis of the influence of breast reconstruction on subsequent development of lymphedema. Plast Reconstr Surg. 2015;136(2):134e–43e. 26218386 10.1097/PRS.0000000000001417

[38] Crosby MA, Card A, Liu J, Lindstrom WA, Chang DW. Immediate breast reconstruction and lymphedema incidence. Plast Reconstr Surg. 2012;129(5):789e–95e. 10.1097/PRS.0b013e31824a2ab1. 22544109 10.1097/PRS.0b013e31824a2ab1

[39] Gunnoo N, Ebelin M, Arrault M, Vignes S. Impact of carpal tunnel syndrome surgery on women with breast cancer-related lymphedema. Breast Cancer Res Treat. 2015;152(3):683–6. 10.1007/s10549-015-3500-6. 26187406 10.1007/s10549-015-3500-6

[40] Schwartz Z, Zalneraitis BH, Milam BP, Warhola MG, Gasbarro G, Galvin JW. Uncomplicated arthroscopic rotator cuff repair in chronic ipsilateral upper extremity lymphedema: a case report. JBJS Case Connect. 2020;10(4):e20.00290. 10.2106/jbjs.Cc.20.00290. 33512918 10.2106/JBJS.CC.20.00290

[41] Ribeiro Pereira ACP, Koifman RJ, Bergmann A. Incidence and risk factors of lymphedema after breast cancer treatment: 10 years of follow-up. Breast. 2017;36:67–73. 10.1016/j.breast.2017.09.006. 28992556 10.1016/j.breast.2017.09.006

[42] Shahpar H, Atieh A, Maryam A, et al. Risk factors of lymphedema in breast cancer patients. Int J Breast Cancer. 2013;2013: 641818. 10.1155/2013/641818. 23862068 10.1155/2013/641818 PMC3687507

[43] Li M, Guo J, Zhao R, Gao J-N, Li M, Wang L-Y. Sun-burn induced upper limb lymphedema 11 years following breast cancer surgery: a case report. World J Clin Cases. 2022;10(32):11987. 36405268 10.12998/wjcc.v10.i32.11987 PMC9669845

[44] Bloomquist K, Krustrup P, Fristrup B, et al. Effects of football fitness training on lymphedema and upper-extremity function in women after treatment for breast cancer: a randomized trial. Acta Oncol. 2021;60(3):392–400. 10.1080/0284186x.2020.1868570. 33423594 10.1080/0284186X.2020.1868570

[45] Showalter SL, Brown JC, Cheville AL, Fisher CS, Sataloff D, Schmitz KH. Lifestyle risk factors associated with arm swelling among women with breast cancer. Ann Surg Oncol. 2013;20(3):842–9. 10.1245/s10434-012-2631-9. 23054109 10.1245/s10434-012-2631-9 PMC4122425

[46] Bloomquist K, Karlsmark T, Christensen KB, Adamsen L. Heavy resistance training and lymphedema: prevalence of breast cancer-related lymphedema in participants of an exercise intervention utilizing heavy load resistance training. Acta Oncol. 2014;53(2):216–25. 10.3109/0284186x.2013.844356. 24195690 10.3109/0284186X.2013.844356

[47] Ugur S, Arıcı C, Yaprak M, et al. Risk factors of breast cancer-related lymphedema. Lymphat Res Biol. 2013;11(2):72–5. 23772716 10.1089/lrb.2013.0004 PMC3685313

[48] Askary ZM, Elshazly M. Addition of local cryotherapy for treatment of post-mastectomy lymphedema. Lymphology. 2022;55(2):70–6. 36170581

[49] Czerniec SA, Ward LC, Kilbreath SL. Breast cancer-related arm lymphedema: fluctuation over six months and the effect of the weather. Lymphat Res Biol. 2016;14(3):148–55. 10.1089/lrb.2015.0030. 27266807 10.1089/lrb.2015.0030

[50] Phillips J, Witt S, Piller N, Gordon S. Seasonal variation in upper limb size, volume, fluid distribution, and lymphedema diagnosis, following breast cancer treatment. Lymphat Res Biol. 2023;21(4):351–8. 10.1089/lrb.2022.0017. 36812466 10.1089/lrb.2022.0017

[51] Koelmeyer LA, Gaitatzis K, Dietrich MS, et al. Risk factors for breast cancer-related lymphedema in patients undergoing 3 years of prospective surveillance with intervention. Cancer. 2022;128(18):3408–15. 10.1002/cncr.34377. 35797441 10.1002/cncr.34377 PMC9542409

[52] Ward LC, Battersby KJ, Kilbreath SL. Airplane travel and lymphedema: a case study. Lymphology. 2009;42(3):139–45. 19927904

[53] Arinaga Y, Piller N, Sato F, et al. The 10-min holistic self-care for patients with breast cancer-related lymphedema: pilot randomized controlled study. Tohoku J Exp Med. 2019;247(2):139–47. 10.1620/tjem.247.139. 30799328 10.1620/tjem.247.139

[54] Du X, Li Y, Fu L, et al. Strategies in activating lymphatic system to promote lymph flow on lymphedema symptoms in breast cancer survivors: a randomized controlled trial. Front Oncol. 2022;12:1015387. 10.3389/fonc.2022.1015387. 36353530 10.3389/fonc.2022.1015387 PMC9638430

[55] Jeffs E, Wiseman T. Randomised controlled trial to determine the benefit of daily home-based exercise in addition to self-care in the management of breast cancer-related lymphoedema: a feasibility study. Support Care Cancer. 2013;21(4):1013–23. 10.1007/s00520-012-1621-6. 23073712 10.1007/s00520-012-1621-6

[56] Ligabue MB, Campanini I, Veroni P, Cepelli A, Lusuardi M, Merlo A. Efficacy of self-administered complex decongestive therapy on breast cancer-related lymphedema: a single-blind randomized controlled trial. Breast Cancer Res Treat. 2019;175(1):191–201. 10.1007/s10549-019-05136-9. 30712198 10.1007/s10549-019-05136-9

[57] Paskett ED, Le-Rademacher J, Oliveri JM, et al. A randomized study to prevent lymphedema in women treated for breast cancer: CALGB 70305 (Alliance). Cancer. 2021;127(2):291–9. 10.1002/cncr.33183. 33079411 10.1002/cncr.33183 PMC7790907

[58] Temur K, Kapucu S. The effectiveness of lymphedema self-management in the prevention of breast cancer-related lymphedema and quality of life: a randomized controlled trial. Eur J Oncol Nurs. 2019;40:22–35. 10.1016/j.ejon.2019.02.006. 31229204 10.1016/j.ejon.2019.02.006

[59] Fu MR, Axelrod D, Guth AA, et al. Proactive approach to lymphedema risk reduction: a prospective study. Ann Surg Oncol. 2014;21(11):3481–9. 10.1245/s10434-014-3761-z. 24809302 10.1245/s10434-014-3761-z PMC4163073

[60] Ochalek K, Gradalski T, Szygula Z. Five-year assessment of maintenance combined physical therapy in postmastectomy lymphedema. Lymphat Res Biol. 2015;13(1):54–8. 10.1089/lrb.2014.0027. 25525902 10.1089/lrb.2014.0027

[61] Cansız G, Arıkan Dönmez A, Kapucu S, Borman P. The effect of a self-management lymphedema education program on lymphedema, lymphedema-related symptoms, patient compliance, daily living activities and patient activation in patients with breast cancer-related lymphedema: a quasi-experimental study. Eur J Oncol Nurs. 2022;56: 102081. 10.1016/j.ejon.2021.102081. 34875398 10.1016/j.ejon.2021.102081

[62] Liu F, Li F, Fu MR, et al. Self-management strategies for risk reduction of subclinical and mild stage of breast cancer-related lymphedema: a longitudinal, quasi-experimental study. Cancer Nurs. 2021;44(6):E493-e502. 10.1097/ncc.0000000000000919. 34694088 10.1097/NCC.0000000000000919

[63] Imamoğlu N, Karadibak D, Ergin G, Yavuzşen T. The effect of education on upper extremity function in patients with lymphedema after breast cancer treatments. Lymphat Res Biol. 2016;14(3):142–7. 10.1089/lrb.2015.0010. 27266576 10.1089/lrb.2015.0010

[64] Gençay Can A, Ekşioğlu E, Çakçı FA. Early detection and treatment of subclinical lymphedema in patients with breast cancer. Lymphat Res Biol. 2019;17(3):368–73. 10.1089/lrb.2018.0033. 30543479 10.1089/lrb.2018.0033

[65] Brophy L. A review of the literature related to limb precautions after lymph node dissection. Number 1/February 2022. 2022;26(1):86–92. 10.1188/22.CJON.86-92 35073289

[66] Hadjistyllis M, Soni A, Hunter-Smith DJ, Rozen WM. A systematic review of the complications of skin puncturing procedures in the upper limbs of patients that have undergone procedures on the axilla or breast. Ann Transl Med. 2023. 10.21037/atm-2. 39118962 10.21037/atm-23-1400 PMC11304417

[67] Jørgensen MG, Toyserkani NM, Hansen FG, Bygum A, Sørensen JA. The impact of lymphedema on health-related quality of life up to 10 years after breast cancer treatment. npj Breast Cancer. 2021;7(1):70. 10.1038/s41523-021-00276-y. 34075045 10.1038/s41523-021-00276-y PMC8169644

[68] Perdomo M, Davies C, Levenhagen K, Ryans K, Gilchrist L. Patient education for breast cancer-related lymphedema: a systematic review. J Cancer Surviv. 2023;17(2):384–98. 36207626 10.1007/s11764-022-01262-4 PMC9546750

[69] McDuff S, Skolny M, Horick N, Miller C, Warren L, Taghian A. Timing of lymphedema following treatment for breast cancer: when are patients most at risk? Int J Radiat Oncol Biol Phys. 2016;96(2):S207. 10.1016/j.ijrobp.2018.08.036 PMC6524147 30165125

